# Active Microrobots for Dual Removal of Biofilms via Chemical and Physical Mechanisms

**DOI:** 10.1021/acsami.4c18360

**Published:** 2025-01-02

**Authors:** Xia Peng, Cagatay M. Oral, Mario Urso, Martina Ussia, Martin Pumera

**Affiliations:** † Future Energy and Innovation Laboratory, Central European Institute of Technology, Brno University of Technology, Purkynova 123, 61200 Brno, Czech Republic; ‡ Department of Medical Research, China Medical University Hospital, China Medical University, No. 91 Hsueh-Shih Road, TW-40402 Taichung, Taiwan; § Advanced Nanorobots & Multiscale Robotics Laboratory, Faculty of Electrical Engineering and Computer Science, VSB—Technical University of Ostrava, 17. Listopadu 2172/15, 70800 Ostrava, Czech Republic

**Keywords:** micromotors, microrobots, photocatalysis, magnetically driven, biofilm, collective motion

## Abstract

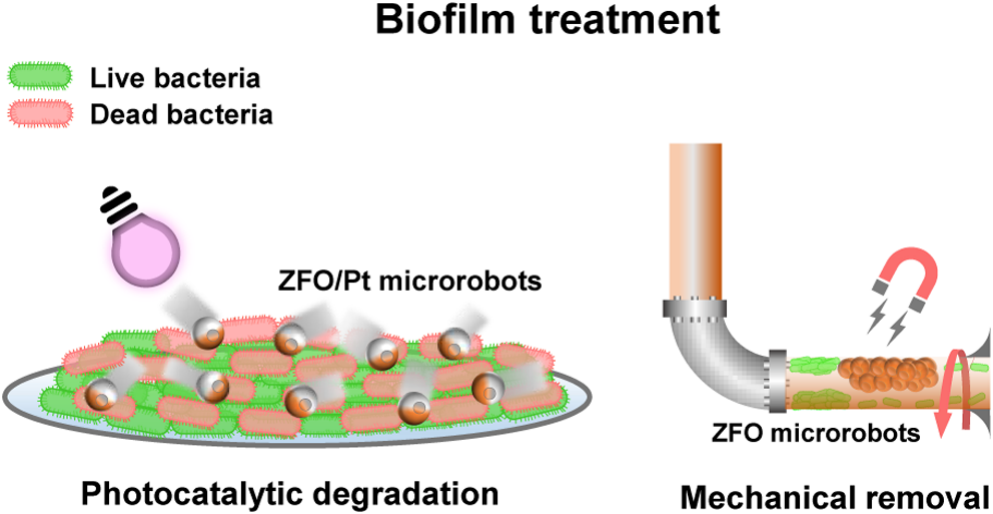

Bacterial biofilms are complex multicellular communities that adhere firmly to solid surfaces. They are widely recognized as major threats to human health, contributing to issues such as persistent infections on medical implants and severe contamination in drinking water systems. As a potential treatment for biofilms, this work proposes two strategies: (i) light-driven ZnFe_2_O_4_ (ZFO)/Pt microrobots for photodegradation of biofilms and (ii) magnetically driven ZFO microrobots for mechanical removal of biofilms from surfaces. Magnetically driven ZFO microrobots were realized by synthesizing ZFO microspheres through a low-cost and large-scale hydrothermal synthesis, followed by a calcination process. Then, a Pt layer was deposited on the surface of the ZFO microspheres to break their symmetry, resulting in self-propelled light-driven Janus ZFO/Pt microrobots. Light-driven ZFO/Pt microrobots exhibited active locomotion under UV light irradiation and controllable motion in terms of “stop and go” features. Magnetically driven ZFO microrobots were capable of maneuvering precisely when subjected to an external rotating magnetic field. These microrobots could eliminate Gram-negative *Escherichia coli* (*E. coli*) biofilms through photogenerated reactive oxygen species (ROS)-related antibacterial properties in combination with their light-powered active locomotion, accelerating the mass transfer to remove biofilms more effectively in water. Moreover, the actuation of magnetically driven ZFO microrobots allowed for the physical disruption of biofilms, which represents a reliable alternative to photocatalysis for the removal of strongly anchored biofilms in confined spaces. With their versatile characteristics, the envisioned microrobots highlight a significant potential for biofilm removal with high efficacy in both open and confined spaces, such as the pipelines of industrial plants.

## 1 Introduction

The emergence of infectious diseases has a strong connection with the proliferation of bacterial biofilms, intricate three-dimensional structures composed of microorganisms, and extracellular polymeric substances (EPS).^1,2^ The process of biofilm formation unfolds through several stages.^3^ Initially, planktonic cells attach on a surface, followed by rapid multiplication and the formation of a protective EPS matrix. This EPS matrix provides the structural foundation for the biofilm’s architecture, accommodating bacterial cells. Moreover, it grants crucial adhesion capabilities to surfaces and enhances resistance against environmental interferences, such as antibiotics and mechanical forces.^4−6^ Beyond the well-documented implications of biofilms for recurrent infections and various medical complications, such as their adhesion to implants and catheters,^7−9^ biofilm formation presents a multifaceted challenge when extended to industrial pipelines and water distribution systems.^10−12^ This issue is particularly pronounced in industrial water-cooling plants and municipal drinking water networks, which have emerged as a significant threat to public health.^13,14^ Hence, the imperative to secure access to pathogen-free drinking water has become highly critical, emphasizing the importance of implementing effective biofilm control measures. Current disinfection methods, such as UV treatment and chemical disinfectants, face challenges like high energy consumption, harmful byproducts, and the risk of promoting resistant biofilms. Moreover, their effectiveness against emerging pathogens is uncertain and requires further evaluation. Novel technologies have recently emerged as a potential remedy, such as chemical removal strategies based on the utilization of photocatalytic nanoparticles to degrade biofilm or physical removal strategies based on the mechanical disruption of biofilm.^15^ These approaches are capable of concurrently targeting the EPS matrix and dormant bacterial cells, holding particular promise for biofilm treatment.^16−19^

In recent years, there has been a remarkable surge in research and development efforts aimed at leveraging microrobots as innovative tools for biofilm eradication.^20−22^ Microrobots are miniature, self-propelled structures that can navigate within complex environments, making them particularly promising for tackling the challenges posed by biofilms.^15,23,24^ One of the pioneering breakthroughs in this field involves the utilization of photocatalytic microrobots capable of propelling themselves under light irradiation.^25−27^ Photocatalytic microrobots can produce highly concentrated reactive oxygen species (ROS) to target biofilm matrices, disrupting their structural integrity, and subsequently making them more susceptible to removal.^28,29^ For example, we introduced a novel concept involving Pt-tubes having a biocompatible TiO_2_ coating to harness light-induced ROS production, which plays a pivotal role in removing dental biofilm.^30^ Alternatively, Ussia et al. demonstrated an effective strategy to eradicate biofilms from solid surfaces by employing light-driven Ag-doped ZnO microrobots, which boost antibiofilm efficacy by increasing ROS production through the catalytic reaction of ZnO and enhancing the diffusion of antimicrobial nanosilver.^10^ Beyond the utilization of photocatalytic materials, microrobots composed of magnetic materials also represent a promising and innovative approach to address biofilm-related challenges by offering a targeted and minimally invasive form of biofilm disruption.^31−33^ When subjected to an external magnetic field, these microrobots demonstrate controlled actuation, which enables them to be maneuvered precisely within biofilm matrices, regardless of the complexity of the biofilm’s architecture.^34,35^ For instance, catalytic antimicrobial robots (CARs) with dual catalytic–magnetic functionality using iron oxide nanoparticles (NPs) were designed to kill, break down, and remove biofilms in a controlled manner. 3D-molded CARs, shaped like vanes or helicoids, targeted specific tasks in enclosed domains by eliminating biofilms and killing bacteria simultaneously.^36^ Similarly, Dong et al. introduced magnetic microswarms with navigation capabilities that effectively eliminated targeted biofilms in both open and confined environments through a synergistic combination of the Fenton reaction and physical disruption.^37^ The precise control and adaptability of magnetic microrobots, along with their ability to operate within the complex environment of biofilms, make them a promising tool for solving biofilm-related issues.^38−40^ Although the different propulsion modes based on one material may provide advanced strategies applicable in daily scenarios associated with biofilms, only a few examples of such microrobots have been reported until now.

**Scheme 1:**
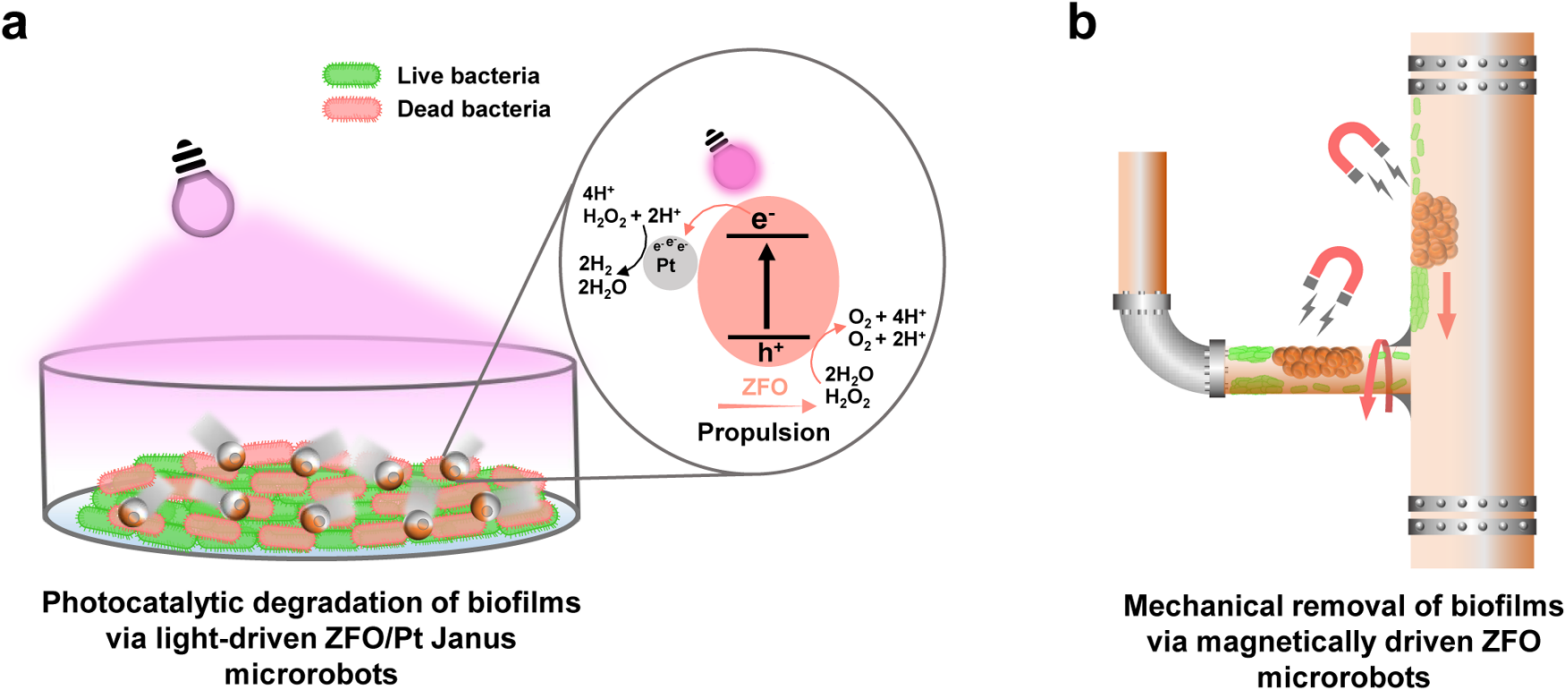
Light-Driven and Magnetic ZFO-Based Microrobots for Effective Biofilm Treatment (a) Light-driven ZFO/Pt microrobots eliminate Gram-negative *E. coli* biofilms via photogenerated ROS-related antibacterial properties. (b) Magnetically driven ZFO microrobots are capable of mechanical removal of biofilms under manipulation by a permanent magnet.

Here, we present ZnFe_2_O_4_ (ZFO)-based microrobots for biofilm treatment from solid surfaces. Previous studies faced challenges in controlling ZnO-based microrobots due to their lack of magnetic properties while Fe_3_O_4_ nanoparticles, despite their superparamagnetism, exhibited weak photocatalytic performance due to a narrow band gap.^10,36,37^ In this work, we address these limitations by utilizing ZFO, which combines both photocatalytic and magnetic properties in one material to enhance the removal of bacterial biofilm through chemical and physical mechanisms. In particular, compared to Fe_2_O_3_, ZFO shows stronger magnetic properties, allowing easy collection of the microrobots using a permanent magnet.^41,42^ This capability not only enables enhanced reusability but also facilitates the efficient isolation of the microrobots from the surrounding environment, thereby minimizing potential interference. Furthermore, ZFO can be considered to have biocompatible characteristics in the environment.^43,44^ Light-driven ZFO particles were synthesized by a facile hydrothermal reaction, followed by a calcination process. Then, to obtain a Janus structure for light-powered self-propulsion, the ZFO microrobots were half-coated with a Pt layer through the sputtering technique, obtaining ZFO/Pt microrobots. When exposed to UV light, electrons (e^–^) in the valence band of ZFO and promoted to the conduction band, leaving holes (h^+^) within the valence band. Electrons migrate from the conduction band to Pt, while holes remain within ZFO, where they contribute to water or H_2_O_2_ decomposition. During this process, it establishes a H^+^ concentration gradient, creating a localized electric field that drives the motion of the microrobots through self-electrophoresis, as depicted in Scheme 1. Their speed values were notably enhanced when subjected to low concentrations of H_2_O_2_ fuel, accompanied by a rapid modulation of their motion/no motion conditions in response to the on/off switch of the UV light source. Due to the intrinsic paramagnetic properties of ZFO microspheres, they can be precisely guided along predefined paths and demonstrate reconfigurable, collective behavior under the influence of an external magnetic field. This functionality is achieved even without the Pt layer, earning them the designation of magnetically driven ZFO microrobots. Gram-negative *Escherichia coli* (*E. coli*) was selected as a model strain for the incubation of biofilm due to its extensive existence in the environment and then treated with light-driven ZFO/Pt microrobots or magnetically driven ZFO microrobots. Light-driven ZFO/Pt microrobots showed excellent performance in killing bacteria under UV light irradiation in the presence of a low concentration of H_2_O_2_, whereas the static microrobots demonstrated only minor removal of the biofilm, which further demonstrates the primary contribution of the dynamic locomotion of microrobots in achieving efficient biofilm removal. Furthermore, magnetically driven ZFO microrobots have been substantiated to effectively prevent biofilm formation from both open surfaces and inner parts of glass tubes having varying diameters, demonstrating that magnetic microrobots hold significant potential for biofilm disruption strategies in both open and confined spaces. This concept is schematically illustrated in Scheme 1. The proposed microrobots, having dual functionalities and scalable fabrication possibilities, hold significant promise for biofilm eradication, especially in addressing long-standing challenges in industrial applications.

## 2 Results and Discussion

### 2.1 Fabrication and Characterization of ZFO-Based Microrobots

**Figure 1:**
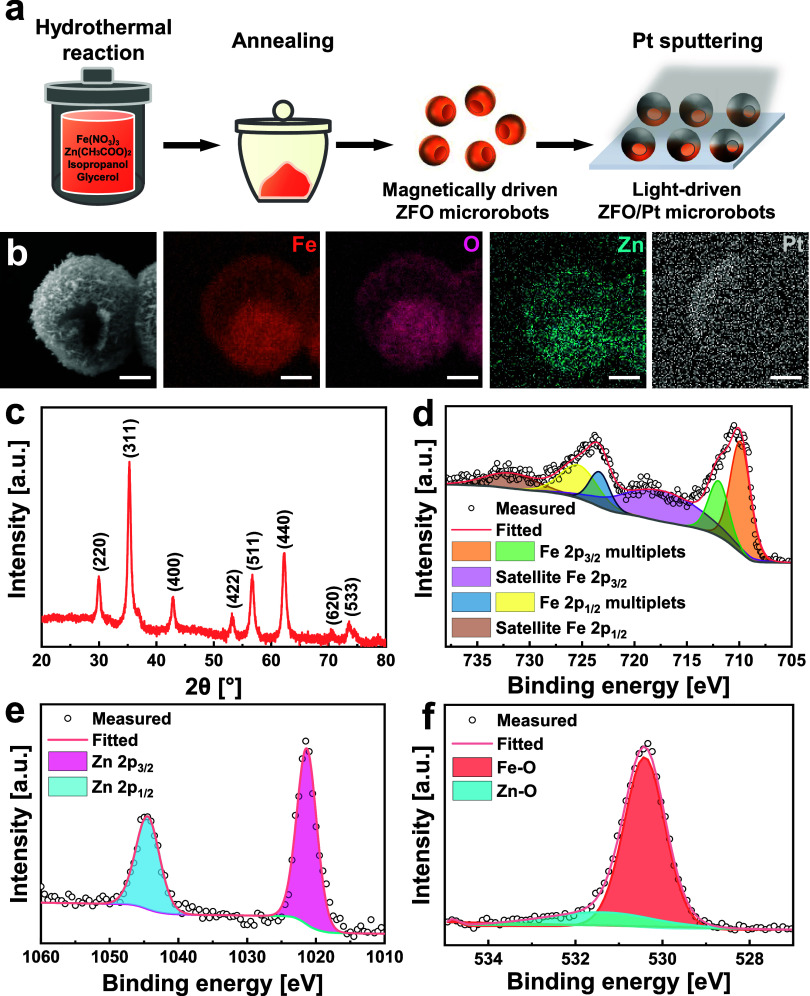
Fabrication and characterization of ZFO-based microrobots. (a) Schematic illustration of the preparation of magnetically driven ZFO microrobots and light-driven ZFO/Pt microrobots. (b) SEM and EDX mapping images of a light-driven ZFO/Pt microrobot showing the distribution of Fe, O, Zn, and Pt elements. Scale bars are 1 μm. (c) XRD spectrum of magnetically driven ZFO microrobots. (d–f) High-resolution XPS spectra of magnetically driven ZFO microrobots for Fe 2p, Zn 2p, and O 1s regions, respectively.

Magnetically driven ZFO microrobots were fabricated by a hydrothermal reaction, followed by a calcination process. Light-driven ZFO/Pt microrobots were prepared by asymmetrical Pt sputtering as schematically shown in Figure 1a, resulting in the half-coating of ZFO microrobots with a Pt layer. The morphology of ZFO-based microrobots was characterized and illustrated by scanning electron microscopy (SEM) images in Figures S1 and 1b. These micrographs display a spherical morphology with a hollow structure and a diameter of approximately 2.5 μm. This structure could be justified by the high temperature reached during the hydrothermal process (180 °C) and the formation of ZnFe-glycolate nuclei due to the solvent’s high surface energy, which is responsible for its tendency to aggregate into a spherical structure, thereby reducing the free energy.^45^ During the calcination process, the ZnFe-glycolate nuclei decompose into ZFO, which eventually results in the formation of hollow ZFO microspheres. In addition, the hollow spheres possess a notably rough surface composed of closely interconnected ZFO nanosheets. Elemental mapping images of ZFO were attained by energy-dispersive X-ray spectroscopy (EDX) to prove their elemental composition and effective deposition of the Pt layer. The EDX images in Figure S1 indicate a uniform distribution of Fe, O, and Zn elements of magnetically driven ZFO microrobots, while Figure 1b demonstrates the asymmetrical Pt layer deposition. To further prove the Janus structure of ZFO/Pt microrobots, SEM measurements using a backscattered electron (BSE) detector were conducted to determine the elemental distribution and clearer contrast between different elements, as shown in Figure S2. The SEM image on the right clearly distinguishes the half-coated Pt layer from the ZFO particles, confirming the Janus structure of the microrobots. Additionally, the EDX images in Figure S2b further validate the Pt layer distribution, which aligns with the SEM observations.

Structural analysis of the microrobots was performed by X-ray diffraction (XRD) within the 2θ range of 20–80°. As depicted in Figure 1c, the XRD pattern reveals consistency between all of the reflection peaks and the standard JCPDS card No. 22–1012, confirming the presence of the spinel ZFO phase.^46^ Specifically, the diffraction peaks at 2θ values of 29.92, 35.28, 36.92, 42.88, 53.16, 56.72, 62.24, and 73.64 can be attributed to the (220), (311), (222), (400), (422), (511), (440), and (533) planes, respectively, which correspond to the cubic phase of spinel ZFO. The absence of any discernible peaks in the spectrum that correspond to ZnO and Fe_2_O_3_ highlights the crystalline purity of the resulting magnetically driven ZFO microrobots.

X-ray photoelectron spectroscopy (XPS) was carried out to investigate the surface composition and chemical states of the ZFO microrobots. The corresponding fitted data for the high-resolution Fe, Zn, and O regions are presented in Figure 1d–f, respectively. Fe 2p spectrum displays peaks at 710.8 (718.7) and 712.9 (725.0) eV binding energies, corresponding to Fe 2p_3/2_ (Fe 2p_1/2_), which are ascribed to tetrahedral and octahedral sites within ZFO, respectively, and signify the presence of Fe^3+^ in the microrobots.^47,48^ In Figure 1e, the Zn 2p spectrum reveals fitting peaks at 1022.1 and 1045.0 eV, corresponding to the binding energy of Zn 2p_1/2_ and Zn 2p_3/2_, respectively, which suggests the presence of Zn^2+^ in the ZnFe_2_O_4_ structure. O 1s spectra exhibit a peak at ∼532 eV, which is attributed to typical lattice oxygen within the structure of Zn–O or Fe–O, while the peak at ∼530 eV designates the presence of adsorbed oxygen on the microrobot’s surface.

### 2.2 Motion Behavior of Light-Driven ZFO/Pt Microrobots

**Figure 2:**
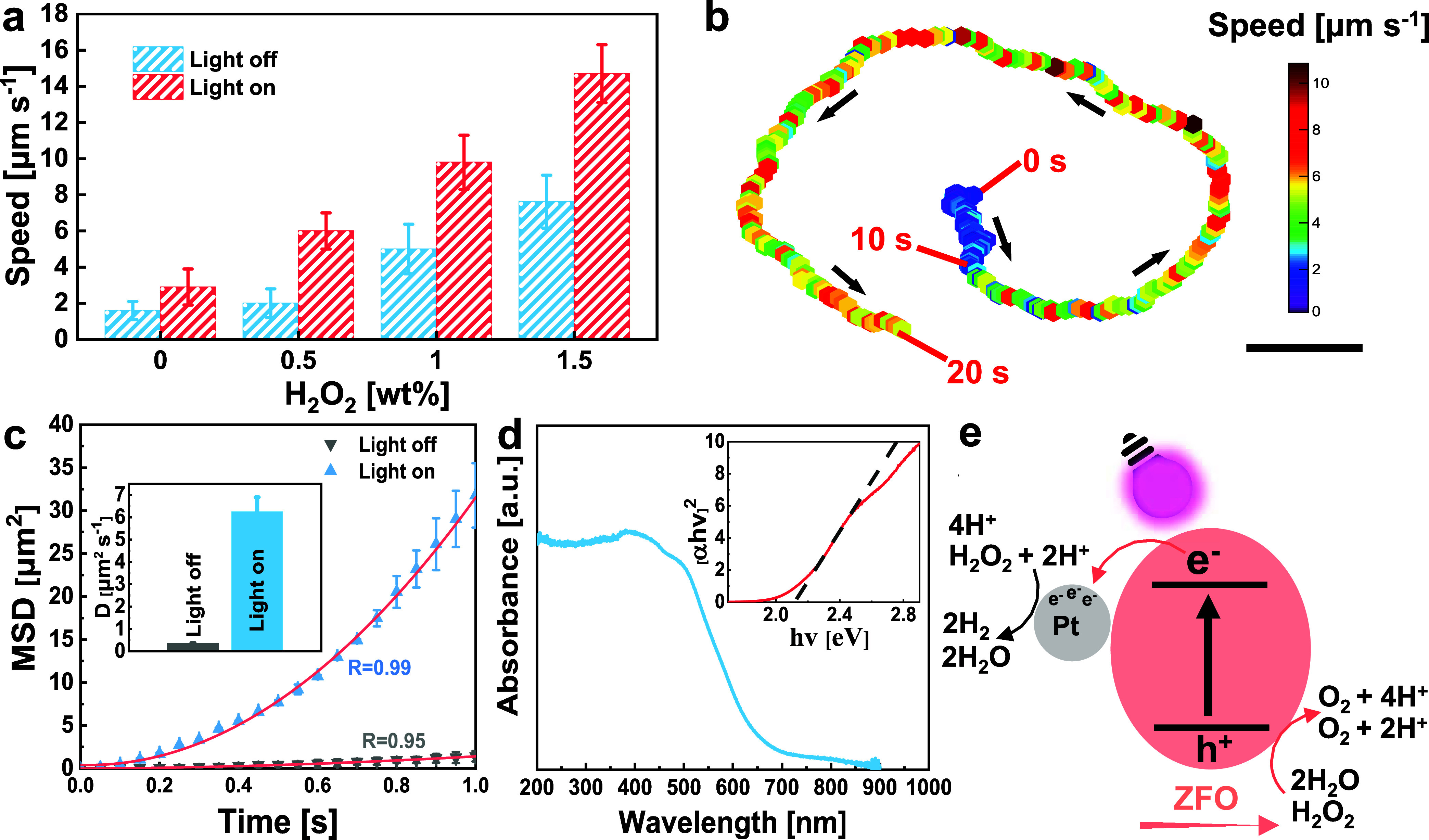
Motion behavior of light-driven ZFO/Pt microrobots. (a) Average speed values of the light-driven ZFO/Pt microrobots at various concentrations of H_2_O_2_ without (light off) or with (light on) UV light irradiation. Error bars represent the standard deviation with *n* = 3 independent replicates. (b) Trajectory and instantaneous speed (color-coded) of a microrobot in 0.5 wt % H_2_O_2_ without (0–10 s) and with (10–20 s) UV light irradiation. Scale bar = 10 μm. Black arrows represent the motion directions of the microrobot. (c) MSD plots of the light-driven ZFO/Pt microrobots in 0.5 wt % H_2_O_2_ without and with UV light irradiation. The inset shows the corresponding diffusion coefficients (*D*) calculated by fitting the MSD plots. (d) UV–vis absorbance spectrum of the light-driven ZFO/Pt microrobots. The inset reports the corresponding Tauc plot. (e) Schematic illustration of the proposed propulsion mechanism of the light-driven ZFO/Pt microrobots.

Motion analysis of light-driven ZFO/Pt microrobots was performed both under UV light illumination and under dark conditions. Different amounts of H_2_O_2_ were eventually employed as a fuel. As can be appreciated from Figure 2a, under UV light exposure, the microrobots exhibited free-fuel motion in water, registering a speed value of about 3 μm s^–1^. The speed of the microrobots further increased up to 9.8 and 14.7 μm s^–1^ by adding 1 and 1.5 wt % of H_2_O_2_, respectively. Differently, in the dark, the microrobots did not show any significant displacement without H_2_O_2_ or in the presence of 0.5 wt % H_2_O_2_. When 1 and 1.5 wt % H_2_O_2_ were introduced under the dark condition, the speed of microrobots registered about 5.1 and 7.6 μm s^–1^, respectively. Therefore, at high H_2_O_2_ concentrations, the microrobots could move, even without UV light irradiation. Figure 2b displays a characteristic trajectory and instantaneous speed (color-coded) of a single microrobot in the dark and under UV light irradiation (Supporting Movie 1). The instantaneous speed is ∼1.5 μm s^–1^ without UV light in the initial 10 s, which is in good agreement with Figure 2a. Afterward, UV light boosted the microrobot speed in a circular trajectory, demonstrating that light irradiation can make a noticeable difference in the speed and trajectory of ZFO/Pt microrobots.

To further analyze the motion behavior of the microrobots under different conditions, a mean squared displacement (MSD) analysis was conducted (Figure 2c). In the presence of 0.5 wt % H_2_O_2_, MSD values of ZFO/Pt microrobots exhibited a linear increase under the dark condition (gray pattern), indicating Brownian motion. However, when UV light was introduced, MSD values followed a parabolic relationship with time. This observation is attributed to the light-driven self-propulsion of ZFO/Pt microrobots.^49^ Based on the analysis of MSD values, diffusion coefficients (*D*) of the microrobots were also calculated (Figure 2c). *D* values significantly increased from 0.4 μm^2^ s^–1^ under dark to 6.3 μm^2^ s^–1^ under UV light irradiation in the presence of 0.5 wt % H_2_O_2_, thus highlighting the light-induced active motion of ZFO/Pt microrobots.

The optical band gap (*E*_g_) for the microrobots was determined based on absorption measurements by UV–vis spectroscopy.^50^ The Tauc plot presented in Figure 2d indicates an *E*_g_ value of 2.1 eV. The potential motion mechanism of light-driven ZFO/Pt microrobots is presented in Figure 2e. ZFO, as an n-type semiconductor with a band gap of 2.1 eV, exhibits light absorption capabilities. When subjected to UV light irradiation, electrons in the semiconductor’s valence band are promoted to the conduction band, thereby leaving vacancies or holes within the valence band.^51^ Electrons migrate from the conduction band to Pt while holes remain confined within the ZFO, where they actively participate in water decomposition reactions as illustrated in Figure 2e.

Notably, the protons (H^+^) generated on the ZFO side are consumed at the Pt side, leading to the production of H_2_. This process establishes a large H^+^ concentration gradient, consequently generating a localized electric field that propels the microrobots through a mechanism known as self-electrophoresis.^52,53^ To highlight the catalytic properties of ZFO/Pt microrobots, a comparison of the catalytic rates between Fe_3_O_4_, ZFO, and ZFO/Pt microrobots, using picric acid (PA) as a degradation model, was conducted (Figure S3). The rate constant, calculated using first-order kinetics, showed a trend of *k* (ZFO/Pt) > *k* (Fe_3_O_4_) > *k* (ZFO), clearly demonstrating the enhanced catalytic efficiency of ZFO/Pt microrobots.

### 2.3 Motion Behavior of Magnetically Driven ZFO Microrobots

**Figure 3:**
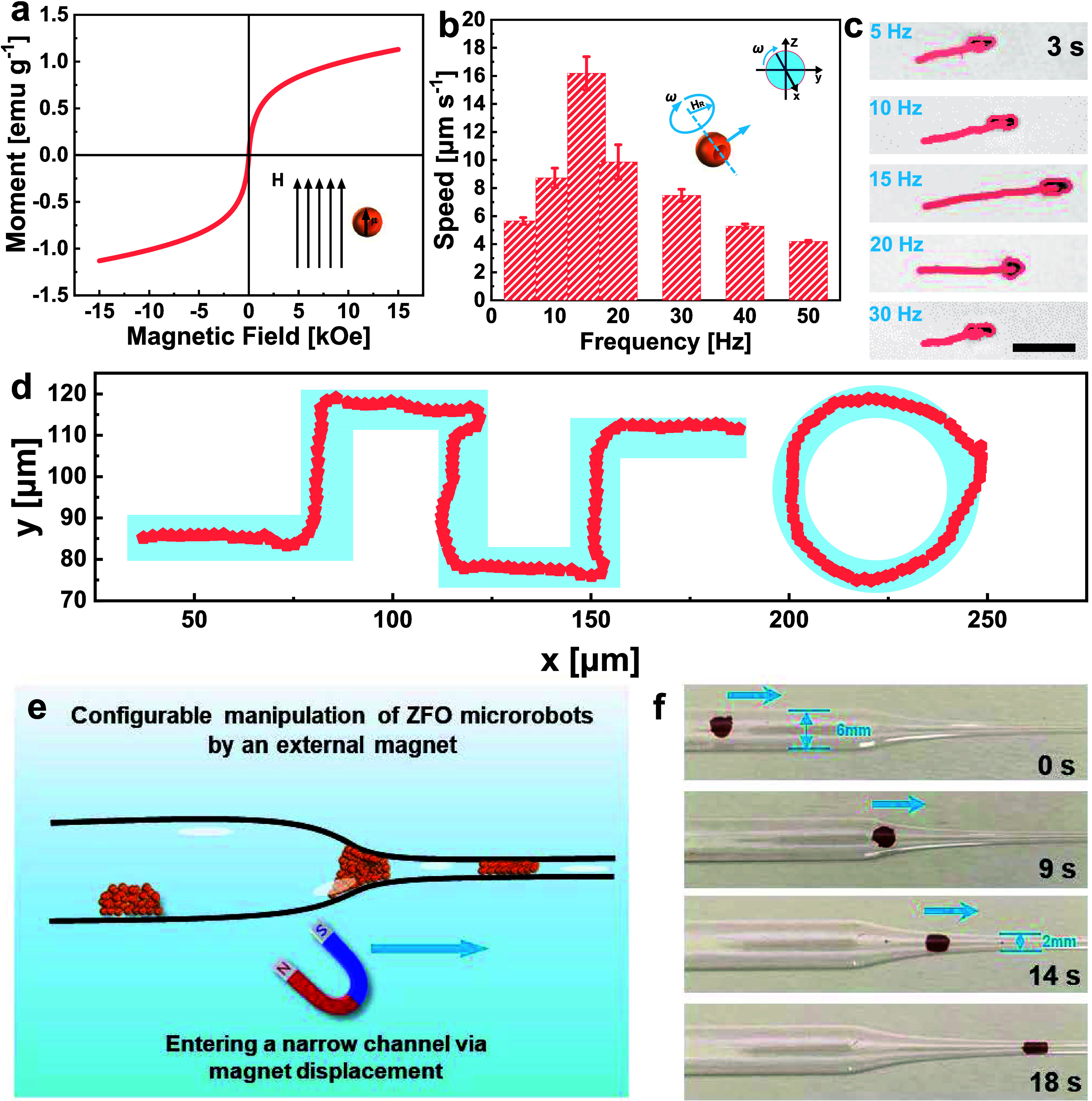
Magnetic motion of ZFO microrobots. (a) Magnetic hysteresis loop of magnetically driven ZFO microrobots. The inset depicts the magnetic dipole moment (**μ**) of a magnetically driven ZFO microrobot and how it aligns with the direction of an externally applied magnetic field (**H**). (b) Average speed values of magnetically driven ZFO microrobots under a transverse rotating magnetic field. Error bars represent the standard deviation with *n* = 10 independent replicates. (c) Trajectories of a magnetically driven ZFO microrobot under a transversal rotating magnetic field (5 mT) at different frequencies (5–30 Hz) for 3 s. (d) Trajectories of a ZFO microrobot along predefined paths under a transversal magnetic field of 5 mT at 15 Hz frequency. (e) Schematic illustration of configuration and external manipulation of a magnetically driven ZFO microrobot agglomerate. (f) Time-lapse digital images of the manipulation of a microrobot agglomerate inside a glass tube, showing its adaptability to the varying tube diameters.

Magnetically driven ZFO microrobots could allow external magnetic control due to the presence of Fe within their structure. To analyze the magnetic properties of the ZFO microrobots, a vibrating sample magnetometer (VSM) was utilized. The magnetic hysteresis loop shown in Figure 3a presents the distinctive characteristics of a paramagnetic material.^54^ The magnetic dipole moment is symmetrically located in the center of the spherical structure. After analysis of the magnetic features, the rolling motion of magnetically driven ZFO microrobots was investigated under the influence of a transverse rotating magnetic field. The magnetic field was kept constant (5 mT) during the experiments, while the frequency was varied from 5 to 50 Hz. As shown in Figure 3b, the average speed of the microrobots increased with the frequency, reaching its peak value of 16.2 μm s^–1^ at 15 Hz, commonly referred to as the “step-out” frequency.^55^ Beyond this frequency, the average speed experienced a notable decrease until 4.3 μm s^–1^ at 50 Hz due to insufficient time to maintain synchronous alignment between the rotation of the magnetic field and the rotation of the magnetically driven ZFO microrobots. The dependency of average speed values on the magnetic field’s frequency also affects the total distance covered by the microrobots. Figure 3c reports micrographs, captured from Supporting Movie 2, of a single microrobot’s trajectory under different frequencies (5–30 Hz) for 3 s. As expected, the longest trajectory is observed at 15 Hz due to the higher speed of magnetically driven ZFO microrobots under this condition.

Magnetically driven ZFO microrobots also enabled precise control of their trajectory, creating intricate patterns, as demonstrated in Figure 3d and Supporting Movie 3. In addition to the manipulation of individual microrobots, the actuation of multiple microrobots and their adaptability to the surrounding environment are of great importance for implementing synchronized tasks, especially in narrow and varying spaces as requested by practical applications, including water remediation in industrial pipelines. To test magnetically driven ZFO microrobots’ abilities in this regard, a glass tube with varying diameters (Figure S4) was selected to simulate their motion behaviors in a real-world setting. Microrobots were injected into the tube’s narrow area as schematically illustrated in Figure 3e. Here, a neodymium–iron–boron (NdFeB) magnet was used to guide the magnetically driven ZFO microrobots instead of the transverse rotating magnetic field. Digital images in Figure 3f demonstrate the displacement of the microrobots from the wide part (6 mm) to the narrow part (2 mm) of the transparent tube in 14 s (Supporting Movie 4). The regulable locomotion performance of magnetically driven ZFO microrobots introduces a pivotal role in undertaking diverse tasks for biomedical applications as well as environmental remediation, such as precise drug delivery, clinical imaging, and the degradation of pollutants at target locations.^25,55−57^ Considering the conventional approach to enable magnetic navigability (obtaining a Janus structure by Ni deposition or incorporating superparamagnetic nanoparticles on particle surfaces), magnetically driven ZFO microrobots present a significant advantage to obtaining magnetic features in a cost-effective and reproducible way due to their intrinsic magnetic properties.^58−60^

### 2.4 Biofilm Treatment by Light-Driven ZFO/Pt Microrobots

**Figure 4:**
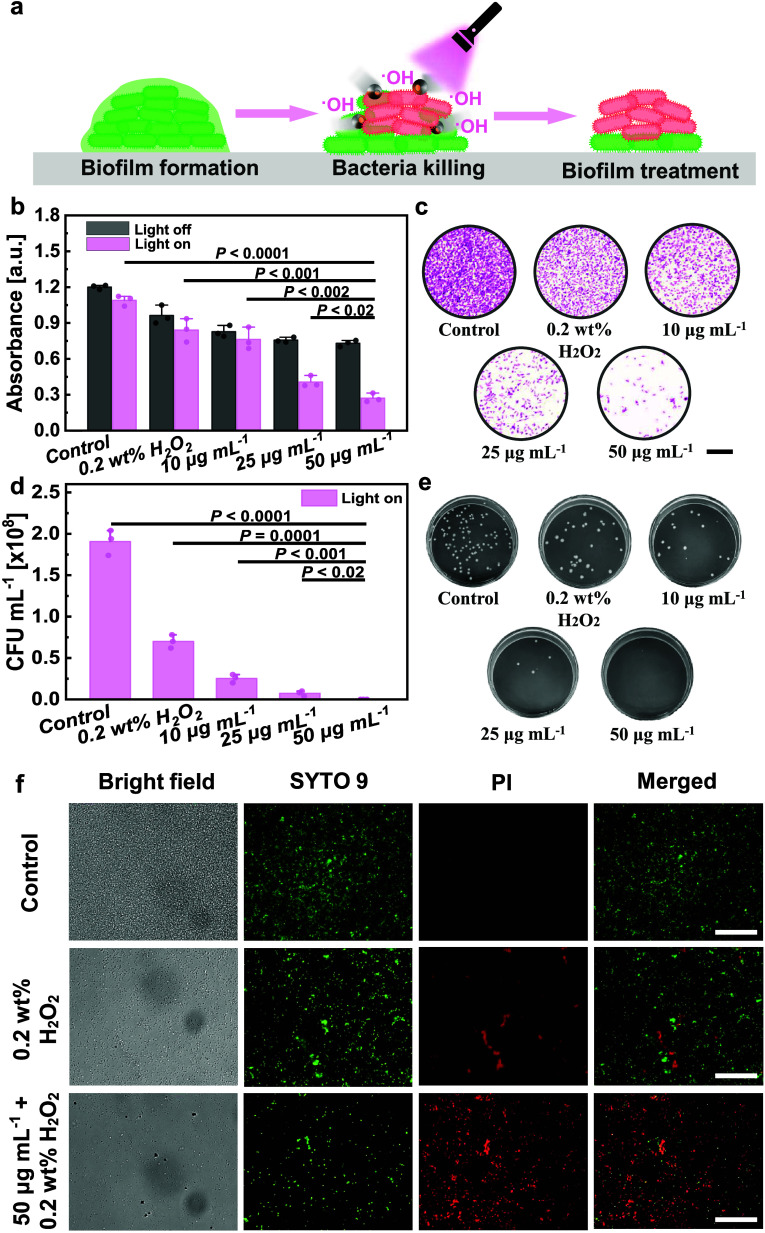
Biofilm removal via light-driven ZFO/Pt microrobots. (a) Schematic illustration of the biofilm removal process in the presence of light-driven ZFO/Pt microrobots, UV light irradiation, and H_2_O_2_ fuel. (b) Absorbance values for stained biofilms treated at different conditions (water, 0.2 wt % H_2_O_2_, or microrobots at 10, 25, and 50 μg mL^–1^ concentrations in the presence of 0.2 wt % H_2_O_2_) without (light off) or with (light on) UV light irradiation for 30 min. (c) Fluorescence images of stained living bacteria after the treatments under different conditions. Scale bar is 10 μm. (d) CFU counts for different groups (water, 0.2 wt % H_2_O_2_, or microrobots at 10, 25, and 50 μg mL^–1^ concentrations in the presence of 0.2 wt % H_2_O_2_) under UV light irradiation for 30 min and (e) the corresponding digital images. (f) LIVE/DEAD assay for biofilms treated for 30 min under UV light irradiation at different conditions (water, 0.2 wt % H_2_O_2_, microrobots 50 μg mL^–1^ concentration in the presence of 0.2 wt % H_2_O_2_). Scale bars: 100 μm. Error bars represent the standard deviation with *n* = 3 independent replicates.

Because the EPS, which is mainly composed of exopolysaccharides, proteins, lipids, and extracellular DNA (eDNA), protects the microorganism from desiccation, oxidation, antibiotics, etc., effective biofilm treatment is a challenging task.^5,61^ As a potential remedy, light-driven ZFO/Pt microrobots, which can produce ROS under light irradiation, were proposed in this study to chemically disrupt the EPS barrier and further kill the protected bacteria cells. The photocatalytic motion of microrobots mainly contributes to the mixing and distribution of ROS rather than directly removing the biofilm, as depicted in Figure 4a. ROS generation by light-driven ZFO/Pt microrobots under light irradiation and in the presence of low concentrations of H_2_O_2_ (0.2%) was validated by fluorescence measurements. As demonstrated in previous work,^30^ terephthalic acid was employed as a probe molecule, effectively trapping hydroxyl radicals and yielding strongly fluorescent 2-hydroxyterephthalic acid. The proportional increase in fluorescent intensity indicates the efficient generation of hydroxyl radicals by ZFO/Pt microrobots (Figure S5). For biofilm experiments, a strain of Gram-negative *E. coli* was selected to investigate the antibacterial performance of light-driven ZFO/Pt microrobots. *E. coli* represents a widely encountered bacteria that typically resides in the intestines of healthy humans and animals.^62^ On the other hand, some strains of *E. coli* can pose health threats in contaminated water systems when ingested or come into contact with the body, causing diarrhea, nausea, and vomiting. More importantly, some *E. coli* strains have also developed resistance to antibiotics, making their eradication more challenging.^63^

In the experimental setup, the growth of *E. coli* biofilms was performed inside 96-well plates. To minimize the effect of H_2_O_2_ on biofilm removal, the lowest concentration of H_2_O_2_ for microrobots motion was investigated. As displayed in Figure S6, the microrobots still exhibited light-triggered motion compared to the dark condition in 0.2% H_2_O_2_ (Supporting Movie 5). These bacterial biofilms were subsequently subjected to different concentrations of light-driven ZFO/Pt microrobots dispersed in a water solution containing 0.2 wt % H_2_O_2_. Simultaneously, the well plates were exposed to 30 min of UV light irradiation to facilitate photocatalytic motion. The antibiofilm activity was compared with data obtained by using water or 0.2 wt % H_2_O_2_ without the presence of the microrobots, to discern the contribution of UV light exposure and H_2_O_2_ to the removal of bacterial biofilms. Biofilm viability was tested using the Crystal Violet (CV) assay by measuring the absorbance value of the dye at 590 nm for each of the aforementioned experimental conditions. As shown in Figure 4b, the absorbance values significantly decreased with higher concentrations of the microrobots during UV light irradiation (light on). The contribution of UV light irradiation or H_2_O_2_ in the absence of the microrobots reveals their limited effect on *E. coli* eradication, especially compared to the condition involving active microrobots at a concentration of 50 μg mL^–1^. Furthermore, control experiments involving static ZFO/Pt microrobots (light off) demonstrated a partial removal of biofilms through Fenton reactions in the presence of ZFO and H_2_O_2_ fuel. The optical images of CV-stained well plates are reported in Figure S7 and further prove that 50 μg mL^–1^ of microrobots led to a much lighter purple color compared to all other conditions (control, 0.2 wt % H_2_O_2_, 10 and 25 μg mL^–1^), indicating that most of the biofilm had been removed from the surface of the well plate. The effect of the duration of UV light exposure was also investigated by extending the irradiation time to 60 min. As illustrated in Figure S8, the results demonstrate that extended irradiation time has only a minimal impact on biofilm removal, indicating that optimal degradation efficiency is achieved within 30 min.

Optical images in Figures 4c and S9 illustrate that the number of stained living bacteria progressively decreased with the increasing concentrations of microrobots in the presence of H_2_O_2_ and UV light irradiation, which is in agreement with Figure 4b. To support these observations, the antimicrobial properties of the light-driven ZFO/Pt microrobots were further evaluated by calculating colony-forming units (CFU) based on a spread plate method as previously reported.^37^ The results of the experiments performed with microrobot-treated groups were compared to the control groups (UV light irradiation and H_2_O_2_). As demonstrated in Figure 4d,e, the bacterial viability significantly decreased after treatment with the microrobots in the presence of UV light and H_2_O_2_, which supports the previous observations based on CV analysis. Finally, a LIVE/DEAD assay was used to validate the efficacy of light-driven ZFO/Pt microrobots against bacterial biofilms. SYTO 9 and propidium iodide (PI) were utilized to detect live (green) and dead (red) bacteria cells, respectively. These cells were initially treated with light-driven ZFO/Pt microrobots (50 μg mL^–1^) under UV light irradiation in the presence of 0.2 wt % H_2_O_2_. Two control groups (only water and 0.2 wt % H_2_O_2_ without the microrobots) were also analyzed under UV light irradiation. The fluorescence microscopy images shown in Figure 4f demonstrate the promising features of the active microrobots toward effective biofilm removal by the significantly higher amount of dead bacteria for the treatment group and negligible effect of UV light or H_2_O_2_ in the absence of light-driven ZFO/Pt microrobots. The treatment of an older and matured biofilm was also taken into consideration by employing light-driven microrobots. Compared to the 24 h biofilm (Figure S10), the 72 h biofilm also exhibited a significant reduction in bacterial colonies, particularly under the optimal conditions of 50 μg mL^–1^ ZFO/Pt microrobots. However, the persistence of a small number of colonies at the highest microrobot concentration suggests that the catalytic efficiency of the microrobots requires further enhancement to effectively eradicate older and more mature biofilms.

### 2.5 Mechanical Removal of Biofilms by Magnetically Driven ZFO Microrobots

**Figure 5:**
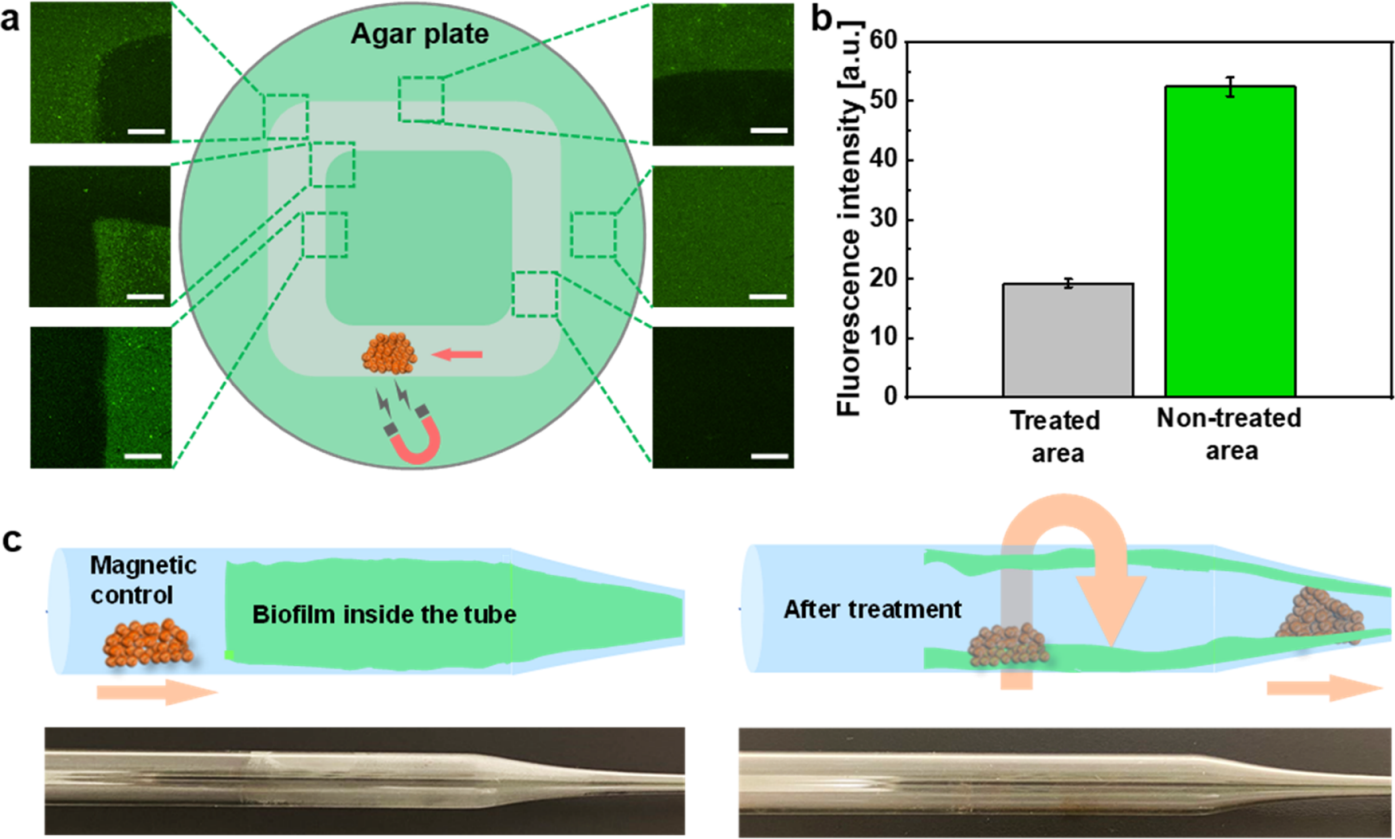
(a) Fluorescent images and (b) the corresponding fluorescence intensity values from different parts of an agar plate after mechanical biofilm disruption achieved by magnetically driven ZFO microrobots under the control of an external magnet. Scale bars are 100 μm. (c) Schematic illustration and digital images of mechanical biofilm disruption in a glass tube.

Different from the chemical disruption of biofilm by light-driven ZFO/Pt microrobots, the magnetic features of magnetically driven ZFO microrobots were utilized for the mechanical removal of the biofilms. For these experiments, a biofilm was successfully grown on an agar plate with 2 days of incubation at 37 °C as shown in Figure S11. An agglomerate of microrobots was then placed on the biofilm surface, followed by external manipulation via a permanent magnet in a predefined trajectory depicted in Figure 5a. Afterward, SYTO 9 was used to detect living bacteria. As evidenced by fluorescence images, mechanically treated areas presented significantly lower amounts of adhered bacteria compared to the areas without any treatment (Figure S12). The fluorescence intensity in Figure 5b decreased significantly after treatment with magnetically driven ZFO microrobots via physical erosion. A further detailed quantitative analysis was determined by comparing the fluorescence intensity in the designated triangular treatment area. From left to right across this region, a considerable decrease in the fluorescence intensity was also detected (Figure S13). Magnetically driven ZFO microrobots also allowed precise positioning for the treatment due to their strong response to external magnetic fields. It is worth mentioning that many areas of biofilm growth are difficult to reach for conventional microparticles, which typically could be observed in narrow plumbing systems or similar scenarios, making it a formidable task to address biofilm contamination.^4^ In response to this requirement, a narrow tube (Figure S4) was used to simulate an enclosed and narrow environment that contains a biofilm on its surface. The magnet allowed easy manipulation of the microrobot agglomerate, leading to the mechanical scratching of the biofilm from the surface, as shown in Figure 5c. These results indicate that the intrinsic magnetic properties of magnetically driven ZFO microrobots can enable targeted biofilm removal even in confined spaces that can be encountered in different daily scenarios. Moreover, the possibility of irradiating UV light after the physical removal process allows for the definitive inactivation of the eradicated bacteria.

## 3 Conclusions

In this study, two effective approaches for the removal of bacterial biofilms were introduced based on light-driven ZFO/Pt microrobots and magnetically driven ZFO microrobots. To utilize the photocatalytic properties of the ZFO microrobots, an asymmetric structure was obtained by the sputtering deposition of Pt on ZFO microspheres synthesized by a hydrothermal method, followed by calcination. Light-driven ZFO/Pt microrobots demonstrated active light-induced motion via self-electrophoresis when exposed to UV light in a controllable way by switching the light on/off. Taking advantage of the paramagnetic properties of magnetically driven ZFO microrobots, they were manipulated collectively with high precision in both open surfaces and enclosed narrow channels. The dual propulsion mechanism of ZFO-based microrobots was utilized for biofilm removal by two strategies. Initially, the photocatalytic activity of light-driven ZFO/Pt microrobots was tested to treat *E. coli* biofilms. The microrobots, together with UV light irradiation and H_2_O_2_ fuel, demonstrated effective biofilm removal due to their active motion and simultaneous ROS production. Additionally, the collective movement of magnetically driven ZFO microrobots under an external magnetic field allowed the mechanical disruption of the biofilm structure inside the surface of a narrow glass tube as a representative example of confined environments. Given that the magnetic actuation of these microrobots could interfere with the stability of the self-electrophoresis mechanism that drives the light-induced motion, synergistic effects of dual motion on biofilm removal remain unconsidered in this work. Instead, the investigation of two motion modes independently for biofilm treatment promotes better insight into their individual contribution. To conclude, these microrobots offer a promising and versatile solution for biofilm treatment in both open surfaces or hard-to-reach confined areas of industrial environments. Owing to the wide variety of micro- and nanorobots that can be created by combining different properties, we focus our efforts on proof-of-concept studies for effective biofilm removal that will lay the foundation for the next generation of intelligent microrobots. In real-world applications, future studies should focus on investigating biofilm elimination using more efficient microrobots in simulated industrial pipelines or drinking water systems.

## 4 Experimental Methods

### 4.1 Synthesis of Magnetically Driven ZFO Microrobots

In a typical synthesis, ZFO microspheres were obtained via a facile hydrothermal process. Briefly, 30 mL of isopropanol and 8 mL of glycerol were mixed in a beaker. Subsequently, 2 mmol of Fe (NO_3_)_3_ and 1 mmol of Zn (CH_3_COO)_2_·2H_2_O were added to the mixture and further stirred for 30 min. The obtained homogeneous mixture was transferred into a Teflon-lined stainless-steel autoclave (50 mL) and kept in a heated oven at 180 °C for 12 h. After the natural cooling of the autoclave until room temperature, the precipitate was obtained by centrifugation at 4500 rpm and washed three times with deionized water and ethanol, followed by drying overnight at 60 °C. Finally, the as-prepared product was further treated via a calcination process in air inside a muffle furnace at 450 °C for 3 h with a heating rate of 5 °C/min. The final product was collected for further utilization.

#### 4.1.1 Fabrication of Light-Driven ZFO/Pt Microrobots

To fabricate the Janus microrobots, the calcinated ZFO microspheres (3 mg mL^–1^) were suspended in DI water and sonicated for 5 min. The suspension was dropped onto glass slides and dried overnight at room temperature. A 30-nm-thick Pt layer was asymmetrically deposited on ZFO microspheres by the sputtering technique. The real-time thickness of the sputtered Pt layer was controlled by a quartz crystal microbalance. Afterward, the microrobots were released from the glass slides using a scalpel.

### 4.2 Material Characterization

The morphology of magnetically driven ZFO and light-driven ZFO/Pt microrobots was assessed by using a Tescan MIRA 3 XMU SEM. EDX mapping analysis was conducted using an Oxford Instruments EDX detector connected to the SEM. XPS spectra were measured with the assistance of a Kratos Analytical Axis Supra instrument. To obtain the fitted data, XPS spectra were analyzed using CASA XPS software. Magnetic hysteresis loop measurements were performed at 300 K in magnetic fields up to 15 kOe using a Quantum Design PPMS VersaLab VSM magnetometer.

### 4.3 Motion Experiments

Microrobot motion was recorded with an inverted microscope (Nikon ECLIPSE TS2R) with a digital camera (Basler acA1920–155uc). The Pt/ZFO microrobots were suspended in DI water and sonicated for 3 min to properly disperse the particles. 0.3 wt % sodium dodecyl sulfate (SDS) was used as a surfactant during the motion experiments. Different concentrations of H_2_O_2_ (0, 0.5, 1, 1.5 wt %) were prepared to assess the speed of microrobots with/without UV light irradiation. The intensity of the light source was fixed at ∼1.5 W cm^–2^. The magnetic motion of the ZFO microrobots was obtained by using a custom-built magnetic setup. The magnetic setup consists of three orthogonal coil pairs in a poly(lactic acid) (PLA) support to generate a transverse rotating magnetic field. Magnetically driven ZFO microrobots were navigated under different intensities of the magnetic field (3 or 5 mT) and different frequencies (5–50 Hz). The recorded videos were analyzed with NIS Elements Advanced Research software.

### 4.4 Biofilm Treatment by ZFO-Based Microrobots

The bacterial strain (*E. coli*, Migula 1895) employed for biofilm experiments was obtained from the Czech Collection of Microorganisms (CCM, Brno, Czech Republic). Initially, bacteria from the stock culture were inoculated onto nutrient agar plates and incubated overnight at 37 °C. Afterward, bacteria were collected in Luria–Bertani (LB) broth and then diluted with LB broth to reach an optical density of 0.15 absorbance units (AU) at 590 nm (OD_590_). Then, the suspended bacteria (200 μL) were seeded into the wells of a 96-well plate. LB broth was refreshed after 1 h and the bacteria were further incubated for 24 h at 37 °C. Following the incubation period, bacterial biofilms were washed twice with phosphate-buffered saline (PBS). The efficacy of the microrobots toward biofilm removal was assessed by their utilization at various concentrations in the range of 10–50 μg mL^–1^. The biofilm removal experiments were conducted in DI water containing 0.2 wt % H_2_O_2_ with or without exposure to UV light irradiation (UV LED at 365 nm, 9 W). Control experiments involved only DI water or H_2_O_2_ without the microrobots. After waiting 30 min, the wells were washed twice with PBS. The presence of biofilm was assessed by using a 1% CV solution. After waiting with CV solution for 15 min, the bacteria were washed twice with PBS. Finally, ethanol was added to dissolve CV and absorbance values were measured at 590 nm using a microplate reader. To further evaluate the efficacy of biofilm killing by the light-driven ZFO/Pt microrobots, CFU assay was conducted. After the treatments, the wells were washed once with PBS. Subsequently, the remaining bacteria were collected by pipet and transferred to centrifuge tubes. The suspension was serially diluted 5 times in PBS. 50 μL of the final bacterial suspension was seeded on agar plates followed by incubation for 24 h at 37 °C. After incubation, the CFUs were counted and averaged. Each experiment was repeated 3 times. Lastly, to obtain fluorescent images, a LIVE/DEAD BacLight bacterial viability kit, involving SYTO-9 and PI, was used according to the manufacturer’s specifications.

## Supporting Information Available

SEM and EDS images of magnetic ZFO microrobots; optical images of the glass tube with varying diameters; fluorescence intensity of 2-hydroxyterephthalic acid; speed of light-driven ZFO/Pt microrobots in 0.2% H_2_O_2_; crystal violet staining of biofilm after treatment; absorbance changes for *E. coli* biofilm with different groups; microscope images of living bacteria after treatment; CFU images for different groups; and fluorescent image of the treated region on the agar plate (PDF)
Motion behavior of ZFO/Pt microrobots with on/off switching of UV light irradiation in 0.5 wt % H_2_O_2_ (Movie 1) (AVI)
Magnetic motion of ZFO microrobots at different frequencies of a transversal rotating magnetic field (Movie 2) (AVI)
Trajectories of a single ZFO microrobot along a predefined path under the control of a transversal rotating magnetic field (Movie 3) (AVI)
Reconfiguration and actuation of the collective microrobots swarm under the control of a permanent magnet (Movie 4) (AVI)
Motion behavior of light-driven ZFO/Pt microrobots in 0.2% H_2_O_2_ (Movie 5) (AVI)

## Supplementary Material

am4c18360_si_001.pdf

am4c18360_si_002.avi

am4c18360_si_003.avi

am4c18360_si_004.avi

am4c18360_si_005.avi

am4c18360_si_006.avi

## References

[ref1] Blair K. M.; Turner L.; Winkelman J. T.; Berg H. C.; Kearns D. B. A Molecular Clutch Disables Flagella in the Bacillus Subtilis Biofilm. Science 2008, 320, 1636–1638. 10.1126/science.1157877. 18566286

[ref2] Mah T. F.; Pitts B.; Pellock B.; Walker G. C.; Stewart P. S.; O’Toole G. A. A Genetic Basis for Pseudomonas Aeruginosa Biofilm Antibiotic Resistance. Nature 2003, 426, 306–310. 10.1038/nature02122. 14628055

[ref3] Arnaouteli S.; Bamford N. C.; Stanley-Wall N. R.; Kovács Á. T. Bacillus Subtilis Biofilm Formation and Social Interactions. Nat. Rev. Microbiol. 2021, 19, 600–614. 10.1038/s41579-021-00540-9. 33824496

[ref4] Liu Y.; Shi L.; Su L.; Van der Mei H. C.; Jutte P. C.; Ren Y.; Busscher H. J. Nanotechnology-Based Antimicrobials and Delivery Systems for Biofilm-Infection Control. Chem. Soc. Rev. 2019, 48, 428–446. 10.1039/C7CS00807D. 30601473

[ref5] Peterson B. W.; He Y.; Ren Y.; Zerdoum A.; Libera M. R.; Sharma P. K.; van Winkelhoff A. J.; Neut D.; Stoodley P.; van der Mei H. C.; Busscher H. J. Viscoelasticity of Biofilms and Their Recalcitrance to Mechanical and Chemical Challenges. FEMS Microbiol. Rev. 2015, 39, 234–245. 10.1093/femsre/fuu008. 25725015 PMC4398279

[ref6] Dieltjens L.; Appermans K.; Lissens M.; Lories B.; Kim W.; Van der Eycken E. V.; Foster K. R.; Steenackers H. P. Inhibiting Bacterial Cooperation Is an Evolutionarily Robust Anti-Biofilm Strategy. Nat. Commun. 2020, 11, 107 10.1038/s41467-019-13660-x. 31919364 PMC6952394

[ref7] Chauhan A.; Ghigo J. M.; Beloin C. Study of in Vivo Catheter Biofilm Infections Using Pediatric Central Venous Catheter Implanted in Rat. Nat. Protoc. 2016, 11, 525–541. 10.1038/nprot.2016.033. 26890680

[ref8] Arciola C. R.; Campoccia D.; Montanaro L. Implant Infections: Adhesion, Biofilm Formation and Immune Evasion. Nat. Rev. Microbiol. 2018, 16, 397–409. 10.1038/s41579-018-0019-y. 29720707

[ref9] Sun M.; Chan K. F.; Zhang Z.; Wang L.; Wang Q.; Yang S.; Chan S. M.; Chiu P. W. Y.; Sung J. J. Y.; Zhang L. Magnetic Microswarm and Fluoroscopy-Guided Platform for Biofilm Eradication in Biliary Stents. Adv. Mater. 2022, 34, 2201888 10.1002/adma.202201888. 35474246

[ref10] Ussia M.; Urso M.; Dolezelikova K.; Michalkova H.; Adam V.; Pumera M. Active Light-Powered Antibiofilm ZnO Micromotors with Chemically Programmable Properties. Adv. Funct. Mater. 2021, 31, 2101178 10.1002/adfm.202101178.

[ref11] Chan S.; Pullerits K.; Keucken A.; Persson K. M.; Paul C. J.; Rådström P. Bacterial Release from Pipe Biofilm in a Full-Scale Drinking Water Distribution System. npj Biofilms Microbiomes 2019, 5, 9 10.1038/s41522-019-0082-9. 30820334 PMC6385293

[ref12] Farh H. M. H.; El Amine Ben Seghier M.; Taiwo R.; Zayed T. Analysis and Ranking of Corrosion Causes for Water Pipelines: A Critical Review. npj Clean Water 2023, 6, 65 10.1038/s41545-023-00275-5.

[ref13] Gomes I. B.; Simões M.; Simões L. C. An Overview on the Reactors to Study Drinking Water Biofilms. Water Res. 2014, 62, 63–87. 10.1016/j.watres.2014.05.039. 24937357

[ref14] Villa K.; Sopha H.; Zelenka J.; Motola M.; Dekanovsky L.; Beketova D. C.; Macak J. M.; Ruml T.; Pumera M. Enzyme-Photocatalyst Tandem Microrobot Powered by Urea for *Escherichia coli* Biofilm Eradication. Small 2022, 18, 2106612 10.1002/smll.202106612. 35122470

[ref15] Urso M.; Ussia M.; Pumera M. Smart Micro- and Nanorobots for Water Purification. Nat. Rev. Bioeng. 2023, 1, 236–251. 10.1038/s44222-023-00025-9. 37064655 PMC9901418

[ref16] Liu Y.; Naha P. C.; Hwang G.; Kim D.; Huang Y.; Simon-Soro A.; Jung H. I.; Ren Z.; Li Y.; Gubara S.; Alawi F.; Zero D.; Hara A. T.; Cormode D. P.; Koo H. Topical Ferumoxytol Nanoparticles Disrupt Biofilms and Prevent Tooth Decay in Vivo via Intrinsic Catalytic Activity. Nat. Commun. 2018, 9, 2920 10.1038/s41467-018-05342-x. 30065293 PMC6068184

[ref17] Chen Z.; Wang Z.; Ren J.; Qu X. Enzyme Mimicry for Combating Bacteria and Biofilms. Acc. Chem. Res. 2018, 51, 789–799. 10.1021/acs.accounts.8b00011. 29489323

[ref18] Benoit D. S. W.; Sims K. R.; Fraser D. Nanoparticles for Oral Biofilm Treatments. ACS Nano 2019, 13, 4869–4875. 10.1021/acsnano.9b02816. 31033283 PMC6707515

[ref19] Hu Y.; Ruan X.; Lv X.; Xu Y.; Wang W.; Cai Y.; Ding M.; Dong H.; Shao J.; Yang D.; Dong X. Biofilm Microenvironment-Responsive Nanoparticles for the Treatment of Bacterial Infection. Nano Today 2022, 46, 101602 10.1016/j.nantod.2022.101602.

[ref20] Wang Z.; Klingner A.; Magdanz V.; Misra S.; Khalil I. S. M. Soft Bio-Microrobots: Toward Biomedical Applications. Adv. Intell. Syst. 2023, 6, 2300093 10.1002/aisy.202300093.

[ref21] Soto F.; Wang J.; Ahmed R.; Demirci U. Medical Micro/Nanorobots in Precision Medicine. Adv. Sci. 2020, 7, 2002203 10.1002/advs.202002203. PMC7610261 33173743

[ref22] Zhang Z.; Wang L.; Chan T. K. F.; Chen Z.; Ip M.; Chan P. K. S.; Sung J. J. Y.; Zhang L. Micro-/Nanorobots in Antimicrobial Applications: Recent Progress, Challenges, and Opportunities. Adv. Healthcare Mater. 2022, 11, 2101991 10.1002/adhm.202101991. 34907671

[ref23] Wu R.; Zhu Y.; Cai X.; Wu S.; Xu L.; Yu T. Recent Process in Microrobots: From Propulsion to Swarming for Biomedical Applications. Micromachines 2022, 13, 1473 10.3390/mi13091473. 36144096 PMC9503943

[ref24] Zhang F.; Zhuang J.; Li Z.; Gong H.; de Ávila B. E. F.; Duan Y.; Zhang Q.; Zhou J.; Yin L.; Karshalev E.; Gao W.; Nizet V.; Fang R. H.; Zhang L.; Wang J. Nanoparticle-Modified Microrobots for in Vivo Antibiotic Delivery to Treat Acute Bacterial Pneumonia. Nat. Mater. 2022, 21, 1324–1332. 10.1038/s41563-022-01360-9. 36138145 PMC9633541

[ref25] Liu D.; Wang T.; Lu Y. Untethered Microrobots for Active Drug Delivery: From Rational Design to Clinical Settings. Adv. Healthcare Mater. 2022, 11, 2102253 10.1002/adhm.202102253. 34767306

[ref26] Rojas D.; Kuthanova M.; Dolezelikova K.; Pumera M. Facet Nanoarchitectonics of Visible-Light Driven Ag_3_PO_4_ Photocatalytic Micromotors: Tuning Motion for Biofilm Eradication. NPG Asia Mater. 2022, 14, 63 10.1038/s41427-022-00409-0.

[ref27] Ussia M.; Urso M.; Kment S.; Fialova T.; Klima K.; Dolezelikova K.; Pumera M. Light-Propelled Nanorobots for Facial Titanium Implants Biofilms Removal. Small 2022, 18, 2200708 10.1002/smll.202200708. 35535477

[ref28] Guix M.; Mayorga-martinez C. C.; Merkoc A. Nano/Micromotors in (Bio) Chemical Science Applications. Chem. Rev. 2014, 114, 6285–6322. 10.1021/cr400273r. 24827167

[ref29] Hu D.; Li H.; Wang B.; Ye Z.; Lei W.; Jia F.; Jin Q.; Ren K. F.; Ji J. Surface-Adaptive Gold Nanoparticles with Effective Adherence and Enhanced Photothermal Ablation of Methicillin-Resistant Staphylococcus Aureus Biofilm. ACS Nano 2017, 11, 9330–9339. 10.1021/acsnano.7b04731. 28806528

[ref30] Villa K.; Viktorova J.; Plutnar J.; Ruml T.; Hoang L.; Pumera M. Chemical Microrobots as Self-Propelled Microbrushes against Dental Biofilm. Cell Rep. Phys. Sci. 2020, 1, 100181 10.1016/j.xcrp.2020.100181.

[ref31] Yuan K.; Jurado-Sánchez B.; Escarpa A. Dual-Propelled Lanbiotic Based Janus Micromotors for Selective Inactivation of Bacterial Biofilms. Angew. Chem., Int. Ed. 2021, 60, 4915–4924. 10.1002/anie.202011617. 33216439

[ref32] Mayorga-Martinez C. C.; Zelenka J.; Klima K.; Mayorga-Burrezo P.; Hoang L.; Ruml T.; Pumera M. Swarming Magnetic Photoactive Microrobots for Dental Implant Biofilm Eradication. ACS Nano 2022, 16, 8694–8703. 10.1021/acsnano.2c02516. 35507525

[ref33] Dong Y.; Wang L.; Zhang Z.; Ji F.; Chan T. K. F.; Yang H.; Chan C. P. L.; Yang Z.; Chen Z.; Chang W. T.; Chan J. Y. K.; Sung J. J. Y.; Zhang L. Endoscope-Assisted Magnetic Helical Micromachine Delivery for Biofilm Eradication in Tympanostomy Tube. Sci. Adv. 2022, 8, eabq8573 10.1126/sciadv.abq8573. 36206344 PMC9544342

[ref34] Zhou H.; Mayorga-Martinez C. C.; Pané S.; Zhang L.; Pumera M. Magnetically Driven Micro and Nanorobots. Chem. Rev. 2021, 121, 4999–5041. 10.1021/acs.chemrev.0c01234. 33787235 PMC8154323

[ref35] Wang L.; Meng Z.; Chen Y.; Zheng Y. Engineering Magnetic Micro/Nanorobots for Versatile Biomedical Applications. Adv. Intell. Syst. 2021, 3, 2000267 10.1002/aisy.202000267.

[ref36] Hwang G.; Paula A. J.; Hunter E. E.; Liu Y.; Babeer A.; Karabucak B.; Stebe K.; Kumar V.; Steager E.; Koo H. Catalytic Antimicrobial Robots for Biofilm Eradication. Sci. Robot. 2019, 4, eaaw2388 10.1126/scirobotics.aaw2388. 31531409 PMC6748647

[ref37] Dong Y.; Wang L.; Yuan K.; Ji F.; Gao J.; Zhang Z.; Du X.; Tian Y.; Wang Q.; Zhang L. Magnetic Microswarm Composed of Porous Nanocatalysts for Targeted Elimination of Biofilm Occlusion. ACS Nano 2021, 15, 5056–5067. 10.1021/acsnano.0c10010. 33634695

[ref38] Sun B.; Sun M.; Zhang Z.; Jiang Y.; Hao B.; Wang X.; Cao Y.; Chan T. K. F.; Zhang L. Magnetic Hydrogel Micromachines with Active Release of Antibacterial Agent for Biofilm Eradication. Adv. Intell. Syst. 2023, 6, 2300092 10.1002/aisy.202300092.

[ref39] Huang S.; Gao Y.; Lv Y.; Wang Y.; Cao Y.; Zhao W.; Zuo D.; Mu H.; Hua Y. Applications of Nano/Micromotors for Treatment and Diagnosis in Biological Lumens. Micromachines 2022, 13, 1780 10.3390/mi13101780. 36296133 PMC9610721

[ref40] Ji H.; Hu H.; Tang Q.; Kang X.; Liu X.; Zhao L.; Jing R.; Wu M.; Li G.; Zhou X.; Liu J.; Wang Q.; Cong H.; Wu L.; Qin Y. Precisely Controlled and Deeply Penetrated Micro-Nano Hybrid Multifunctional Motors with Enhanced Antibacterial Activity against Refractory Biofilm Infections. J. Hazard. Mater. 2022, 436, 129210 10.1016/j.jhazmat.2022.129210. 35739732

[ref41] Urso M.; Ussia M.; Pumera M. Breaking Polymer Chains with Self-Propelled Light-Controlled Navigable Hematite Microrobots. Adv. Funct. Mater. 2021, 91, 2101510 10.1002/adfm.202101510.

[ref42] Urso M.; Ussia M.; Novotný F.; Pumera M. Trapping and detecting nanoplastics by MXene-derived oxide microrobots. Nat. Commun. 2022, 13, 3573 10.1038/s41467-022-31161-2. 35732658 PMC9218121

[ref43] Manjura Hoque S.; Sazzad Hossain Md.; Choudhury S.; Akhter S.; Hyder F. Synthesis and Characterization of ZnFe_2_O_4_ Nanoparticles and its Biomedical Applications. Mater. Lett. 2016, 162, 60–63. 10.1016/j.matlet.2015.09.066. 26549918 PMC4632970

[ref44] Huang Y.; Liang Y.; Rao Y.; Zhu D.; Cao J.; Shen Z.; Ho W.; Lee S. C. Environment-Friendly Carbon Quantum Dots/ZnFe_2_O_4_ Photocatalysts: Characterization, Biocompatibility, and Mechanisms for NO Removal. Environ. Sci. Technol. 2017, 51, 2924–2933. 10.1021/acs.est.6b04460. 28145696

[ref45] F Fang Z.; Zhang L.; Qi H.; Yue H.; Zhang T.; Zhao X.; Chen G.; Wei Y.; Wang C.; Zhang D. Nanosheet Assembled Hollow ZnFe_2_O_4_ Microsphere as Anode for Lithium-Ion Batteries. J. Alloys Compd. 2018, 762, 480–487. 10.1016/j.jallcom.2018.05.259.

[ref46] Köseoğlu Y.; Baykal A.; Toprak M. S.; Gözüak F.; Başaran A. C.; Aktaş B. Synthesis and Characterization of ZnFe_2_O_4_ Magnetic Nanoparticles via a PEG-Assisted Route. J. Alloys Compd. 2008, 462, 209–213. 10.1016/j.jallcom.2007.07.121.

[ref47] Manohar A.; Vijayakanth V.; Kim K. H. Influence of Ca Doping on ZnFe_2_O_4_ Nanoparticles Magnetic Hyperthermia and Cytotoxicity Study. J. Alloys Compd. 2021, 886, 161276 10.1016/j.jallcom.2021.161276.

[ref48] Sundararajan M.; Sukumar M.; Dash C. S.; Sutha A.; Suresh S.; Ubaidullah M.; Al-Enizi A. M.; Raza M. K.; Kumar D. A Comparative Study on NiFe_2_O_4_ and ZnFe_2_O_4_ Spinel Nanoparticles: Structural, Surface Chemistry, Optical, Morphology and Magnetic Studies. Phys. B 2022, 644, 414232 10.1016/j.physb.2022.414232.

[ref49] Khezri B.; Villa K. Hybrid Photoresponsive/Biocatalytic Micro- and Nanoswimmers. Chem. - Asian J. 2022, 17, e202200596 10.1002/asia.202200596. 35785519

[ref50] Villa K.; Novotný F.; Zelenka J.; Browne M. P.; Ruml T.; Pumera M. Visible-Light-Driven Single-Component BiVO_4_ Micromotors with the Autonomous Ability for Capturing Microorganisms. ACS Nano 2019, 13, 8135–8145. 10.1021/acsnano.9b03184. 31283169

[ref51] Peng X.; Urso M.; Pumera M. Photo-Fenton Degradation of Nitroaromatic Explosives by Light-Powered Hematite Microrobots: When Higher Speed Is Not What We Go For. Small Methods 2021, 5, 2100617 10.1002/smtd.202100617. 34927942

[ref52] Lyu X.; Liu X.; Zhou C.; Duan S.; Xu P.; Dai J.; Chen X.; Peng Y.; Cui D.; Tang J.; Ma X.; Wang W. Active, Yet Little Mobility: Asymmetric Decomposition of H_2_O_2_ Is Not Sufficient in Propelling Catalytic Micromotors. J. Am. Chem. Soc. 2021, 143, 12154–12164. 10.1021/jacs.1c04501. 34339185

[ref53] Brooks A. M.; Tasinkevych M.; Sabrina S.; Velegol D.; Sen A.; Bishop K. J. M. Shape-Directed Rotation of Homogeneous Micromotors via Catalytic Self-Electrophoresis. Nat. Commun. 2019, 10, 495 10.1038/s41467-019-08423-7. 30700714 PMC6353883

[ref54] Navidpour A. H.; Fakhrzad M. Photocatalytic and Magnetic Properties of ZnFe_2_O_4_ Nanoparticles Synthesised by Mechanical Alloying. Int. J. Environ. Anal. Chem. 2022, 102, 690–706. 10.1080/03067319.2020.1726331.

[ref55] Lin Z.; Fan X.; Sun M.; Gao C.; He Q.; Xie H. Magnetically Actuated Peanut Colloid Motors for Cell Manipulation and Patterning. ACS Nano 2018, 12, 2539–2545. 10.1021/acsnano.7b08344. 29443501

[ref56] Oral C. M.; Ussia M.; Urso M.; Salat J.; Novobilsky A.; Stefanik M.; Ruzek D.; Pumera M. Radiopaque Nanorobots as Magnetically Navigable Contrast Agents for Localized In Vivo Imaging of the Gastrointestinal Tract. Adv. Healthcare Mater. 2023, 12, 2202682 10.1002/adhm.202202682. 36502367

[ref57] Jurado-Sánchez B.; Wang J. Micromotors for Environmental Applications: A Review. Environ. Sci. Nano 2018, 5, 1530–1544. 10.1039/C8EN00299A.

[ref58] Shen H.; Cai S.; Wang Z.; Ge Z.; Yang W. Magnetically Driven Microrobots: Recent Progress and Future Development. Mater. Des. 2023, 227, 111735 10.1016/j.matdes.2023.111735.

[ref59] Fu D.; Jiang J.; Fu S.; Xie D.; Gao C.; Feng Y.; Liu S.; Ye Y.; Liu L.; Tu Y.; Peng F. Real-Time Micromotor Probe for Immune Neutrophil Activation State. Adv. Healthcare Mater. 2023, 12, 2300737 10.1002/adhm.202300737. 37199571

[ref60] Valdez-Garduño M.; Leal-Estrada M.; Oliveros-Mata E. S.; Sandoval-Bojorquez D. I.; Soto F.; Wang J.; Garcia-Gradilla V. Density Asymmetry Driven Propulsion of Ultrasound-Powered Janus Micromotors. Adv. Funct. Mater. 2020, 30, 2004043 10.1002/adfm.202004043.

[ref61] Muhammad M. H.; Idris A. L.; Fan X.; Guo Y.; Yu Y.; Jin X.; Qiu J.; Guan X.; Huang T. Beyond Risk: Bacterial Biofilms and Their Regulating Approaches. Front. Microbiol. 2020, 11, 928 10.3389/fmicb.2020.00928. 32508772 PMC7253578

[ref62] Wasiński B. Extra-Intestinal Pathogenic *Escherichia Coli* – Threat Connected with Food-Borne Infections. Ann. Agric. Environ. Med. 2019, 26, 532–537. 10.26444/aaem/111724. 31885224

[ref63] Zhang S.; Abbas M.; Rehman M. U.; Wang M.; Jia R.; Chen S.; Liu M.; Zhu D.; Zhao X.; Gao Q.; Tian B.; Cheng A. Updates on the Global Dissemination of Colistin-Resistant *Escherichia Coli*: An Emerging Threat to Public Health. Sci. Total Environ. 2021, 799, 149280 10.1016/j.scitotenv.2021.149280. 34364270

