# Supplementary material for: Active Microrobots for Dual Removal of Biofilms via Chemical and Physical Mechanisms
Source: ACS Appl Mater Interfaces. 2025 Jan 2;17(2):3608–19. doi: 10.1021/acsami.4c18360 (PMC11744513; doi:10.1021/acsami.4c18360)
Supplement: Supplementary file 1 — am4c18360_si_001.pdf [file am4c18360_si_001.pdf]

Active Microrobots for Dual Removal of Biofilms via Chemical and Physical Mechanisms

Xia Peng[a], Cagatay M. Oral[a], Mario Urso[a], Martina Ussia[a], Martin Pumera[a, b, c*]

[a] Future Energy and Innovation Laboratory, Central European Institute of Technology, Brno University of Technology, Purkynova 123, 61200, Brno, Czech Republic

[b] Department of Medical Research, China Medical University Hospital, China Medical University, No. 91 Hsueh-Shih Road, TW-40402 Taichung, Taiwan

[c] Advanced Nanorobots & Multiscale Robotics Laboratory, Faculty of Electrical Engineering and Computer Science, VSB - Technical University of Ostrava, 17. listopadu 2172/15, 70800 Ostrava, Czech Republic

[d] Department of Chemical and Biomolecular Engineering, Yonsei University, 50 Yonsei-ro, Seodaemun-gu, Seoul 03722, Korea

* Corresponding author e-mail: martin.pumera@ceitec.vutbr.cz

Supporting Information

Supplementary Movie 1. The motion behavior of ZFO/Pt microrobots with on/off switching of UV light irradiation in 0.5 wt% H_2O_2.

Supplementary Movie 2. Magnetic motion of ZFO microrobots at different frequencies of a transversal rotating magnetic field.

Supplementary Movie 3. Trajectories of a single ZFO microrobot along a predefined path under the control of a transversal rotating magnetic field.

Supplementary Movie 4. Reconfiguration and actuation of the collective microrobots swarm under the control of a permanent magnet.

Supplementary Movie 5. Motion behavior of light-driven ZFO/Pt microrobots in 0.2% H_2O_2.

Supplementary Figures

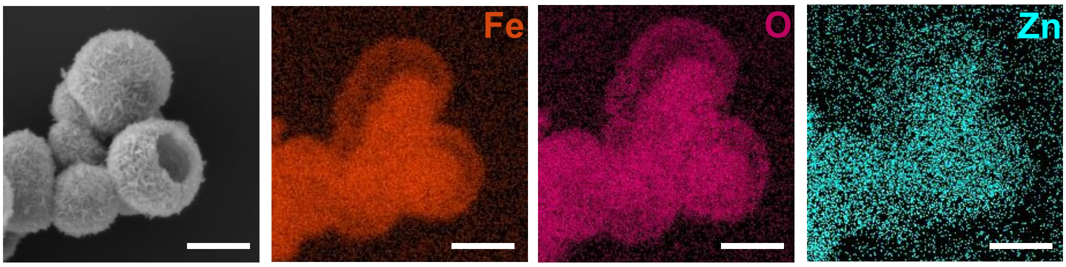

Figure S1. SEM and EDS images of magnetic ZFO microrobots showing the distribution of Fe, O, Zn, and Pt elements. Scale bars are 1 μm.

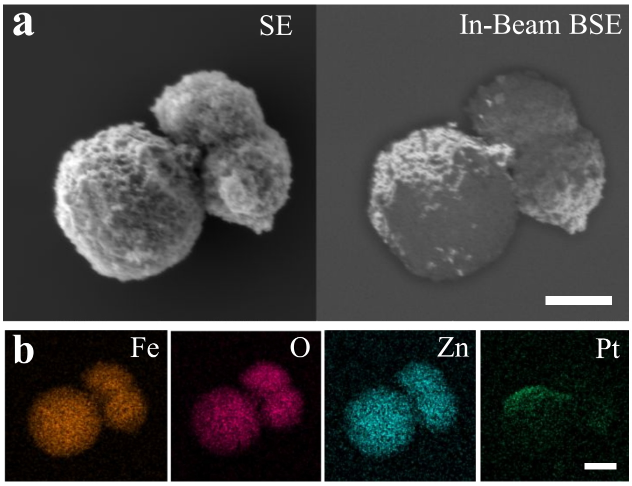

Figure S2. a) SEM images of showing distinct contrast of Pt element on the surface of light-driven ZFO/Pt microrobots and b) Corresponding EDX mapping images showing the distribution of Fe, O, Zn, and Pt elements. Scale bars are 1 μm.

The catalysis rate was evaluated as below according to the first order kinetics model

$$\ln\left(\frac{C_t}{C_0}\right) = -kt \quad (1)$$

where C_0 is the initial concentration of PA, C_t is the concentration of PA at the time t [min], and k [min^{-1}] is the first-order rate constant. The plots of $ln\ (C_t/C_0)$ as a function of t are shown in **Figure S3(a)**. From the linear fitting of these plots, the rate constant k was calculated using Equation (1).

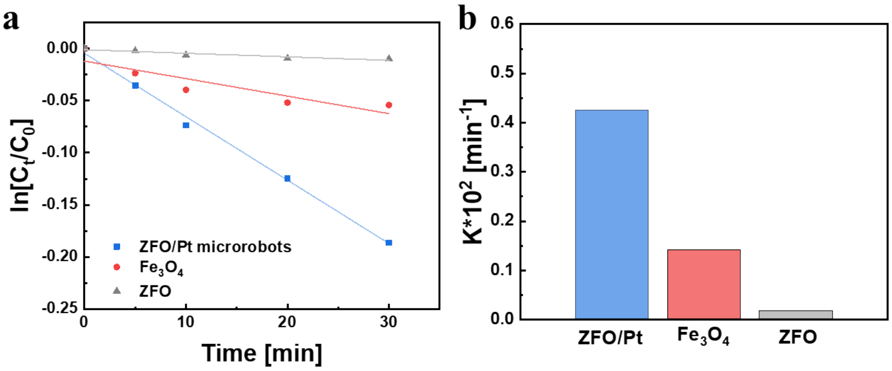

Figure S3. a) $ln\ (C_t/C_0)$ as a function of t and linear fitting for light-driven ZFO/Pt microrobots, ZFO, and Fe_3O_4 under light irradiation in 0.2% H_2O_2. b) The corresponding first-order rate constant (k) values obtained from linear fitting using Equation (1).

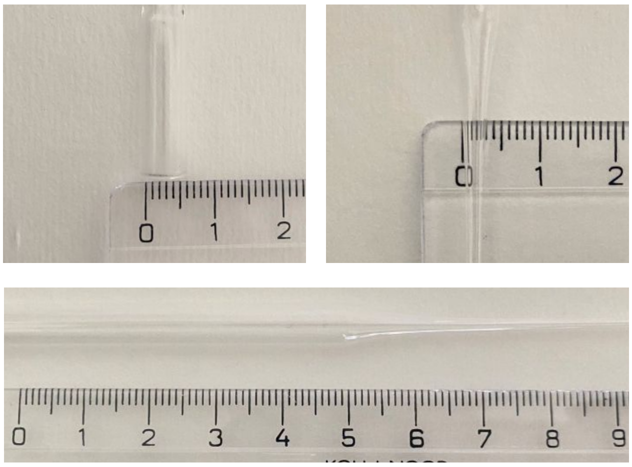

Figure S4. Optical images of the glass tube with varying diameters.

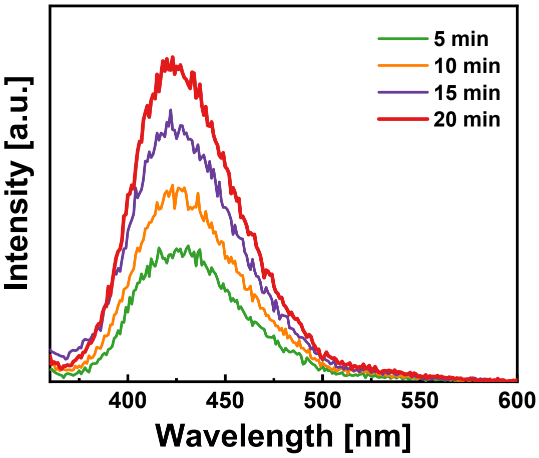

Figure S5. The fluorescence intensity of 2-hydroxyterephthalic acid, arising from the interaction between terephthalic acid and ZFO/Pt microrobots in the presence of 0.2% H_2O_2 solution under UV light lumination.

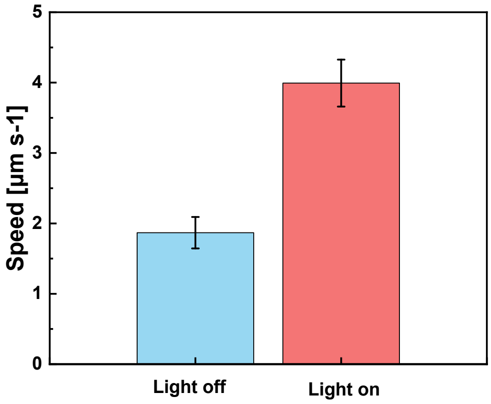

Figure S6. The speed of light-driven ZFO/Pt microrobots in 0.2% H_2O_2 with light on/off conditions.

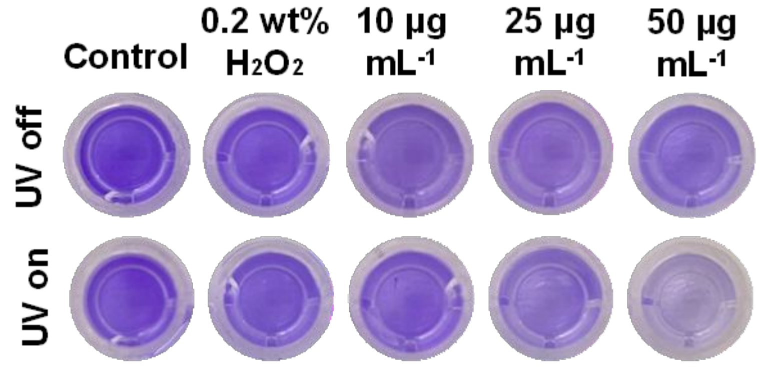

Figure S7. Crystal violet staining of biofilm in dark (top) and biofilm under UV light irradiation for 30 min (bottom), both treated by different groups (biofilm in water; biofilm in 0.2 wt% H_2O_2; biofilm in 0.2 wt% H_2O_2 treated with 10, 25, 50 μg mL^{-1} microrobots).

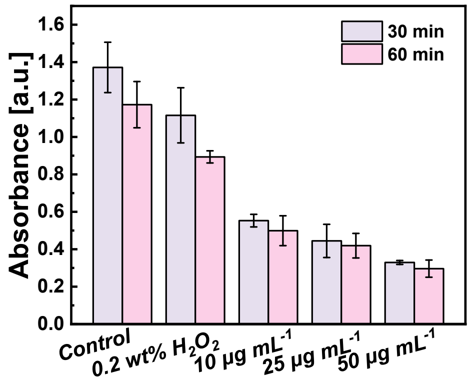

Figure S8. Absorbance changes for *E. coli* biofilm treated by different groups (biofilm in water; biofilm in 0.2 wt% H_2O_2; biofilm in 0.2 wt% H_2O_2 treated with 10, 25, 50 μg mL^{-1} microrobots) under UV light irradiation for 30 and 60 min.

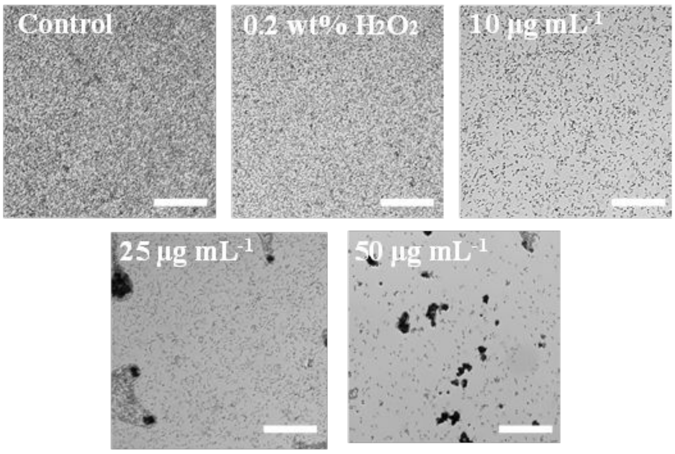

Figure S9. Microscope images of living bacteria after different treatment groups. Scale bars are 10 μm.

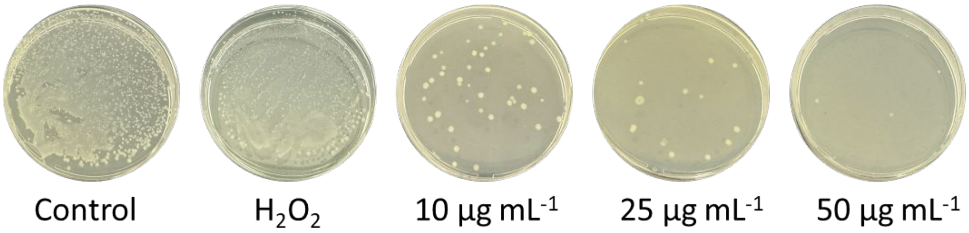

Figure S10. CFU images for different groups (water, 0.2 wt% H_2O_2, or microrobots at 10, 25, 50 µg mL^{-1} concentrations in the presence of 0.2 wt% H_2O_2) under UV light irradiation for 30 min.

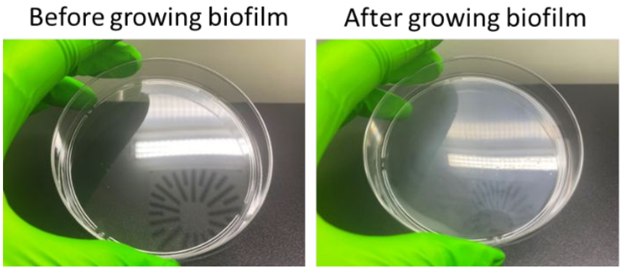

Figure S11. Digital images of *E. coli* biofilm on a solid plate after 24 h incubation at 37 °C.

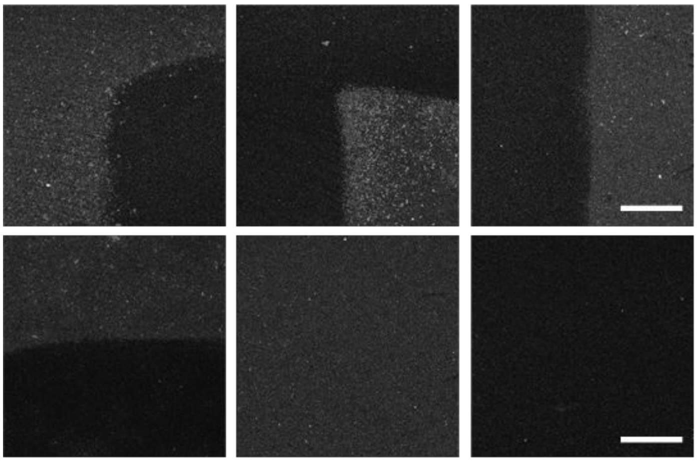

Figure S12. Bright-field optical images according to Figure 5a in the main text, indicating the effective removal of the *E. coli* biofilm incubated on a plate surface by employing magnetic ZFO microrobots under the control of a magnet under the plate. Scale bars are 100 µm.

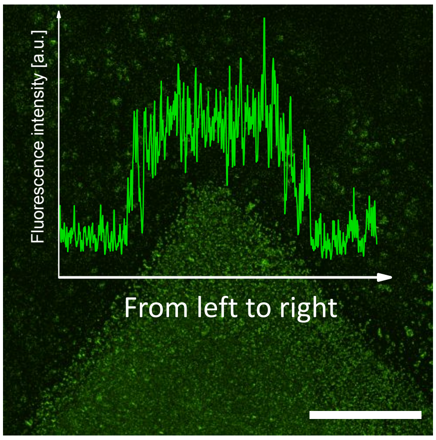

Figure S13. The fluorescent image of the treated region on the agar plate and the responding fluorescence intensity. Scale bars are 100 μm.
